# An analysis on the roles and involvements of different stakeholders in the provision of adolescent sexual and reproductive health services in Southeast Nigeria

**DOI:** 10.1186/s12889-022-14644-1

**Published:** 2022-11-24

**Authors:** Chibuike Agu, Chinyere Mbachu, Ifunanya Agu, Ugenyi Iloabachie, Obinna Onwujekwe

**Affiliations:** 1 Health Policy Research Group, Department of Pharmacology and Therapeutics, College of Medicine, University of Nigeria Enugu-Campus, Enugu, Nigeria; 2 Department of Community Medicine, Alex-Ekwueme Federal University Teaching Hospital, Abakaliki, Ebonyi Nigeria; 3 Department of Community Medicine, College of Medicine, University of Nigeria Enugu-Campus, Enugu, Nigeria; 4 Department of Health Administration and Management, Faculty of Health Sciences and Technology, University of Nigeria Enugu-Campus, Enugu, Nigeria

**Keywords:** Adolescents, ASRH, SRH, Stakeholder mapping, ASRH programs, Ebonyi, Nigeria

## Abstract

**Introduction:**

Sexual and reproductive health of young people involve a lot of stakeholders, traverse different sectors, and cut across all levels of government. For a clearer understanding of the activities of these stakeholders in adolescent sexual and reproductive health (ASRH) services, this paper was designed to explore the positions, attitudes and involvements of government/public institutions and non-governmental organizations (NGOs) in ASRH policy-making processes and implementations in Ebonyi State, Nigeria.

**Methods:**

The evidence was generated from a cross-sectional qualitative study, with data collected through in-depth interviews and focus group discussions from 81 and 59 stakeholders in adolescent sexual and reproductive health, respectively. A mapping tool was used for the stakeholder analysis.

**Results:**

The State Ministry of Health (MOH) was identified as playing a major role in ASRH policy development and so was categorized as a ‘savior’. However, out of nine public institutions, four were categorized as ‘trip wire’ because they had non-supportive attitudes, weak powers and passive interests in ASRH policies and programs. All the NGOs were categorized as ‘friend’, because of their weak, but favorable disposition to ASRH policy-making processes. Regarding the implementation of ASRH programs, most public institutions were categorized as ‘savior’. Similarly, most of the institutions were classified as ‘trip wire’ at the local government level, in relation to ASRH policy development. Only, the offices of traditional rulers/village heads and local government administrative secretaries were regarded as ‘friend’, while the public schools were classified as an ‘acquaintance’. Concerning the implementation of ASRH programs at this level, public secondary schools, the offices of local government administrative secretaries and local government focal persons on ASRH were categorized as ‘savior’, while town union/ward development chairmen were considered ‘friend’. Few stakeholders, including, religious leaders were classified as ‘savior’ regarding engagement with local authorities on ASRH matters.

**Conclusion:**

Although key stakeholders appear to play supportive roles in the implementation of ASRH programs in Ebonyi State, many of the relevant government and non-government institutions are not involved in the policy-making process. There is a need for more intentional and active involvement of relevant stakeholders in policy-making for better ownership and sustainability of ASRH interventions.

## Introduction

The need to address adolescents’ sexual and reproductive health (SRH) is both a global concern and a major challenge for most developing countries [1, 2]. Promoting and protecting the reproductive health of young people enhances their overall wellbeing and development into healthy and productive adulthood [2]. Healthy adolescence is essential for economic growth through rising productivity and prevention of the generational spread of diseases [3]. It is also critical to achieving the Sustainable Development Goals (SDGs), particularly those targeting poverty, health security, education and reducing inequalities [4].

The SRH issues of young people traverse different sectors, such as health, education, information, women, youth development and sports. Similarly, it cuts across all levels of government and civil society, the academia and the private sectors [1]. These stakeholders play various roles in policy making, program development and implementation of SRH interventions for young people [1, 5]. So, the successful provision of SRH information and services to adolescents will depend on these players, including the government as decision-makers, program implementers (schools, health care facilities), and clients/beneficiaries (adolescents) [6].

Adolescents are the only age group in which HIV-related deaths have not declined [7]. They represent a growing population of people living with HIV, making up about 11% of new adult HIV infections worldwide [8]. In addition, the risk of maternal mortality is highest for adolescent girls under 15 years old, and complications in pregnancy and childbirth are higher among adolescents aged between 10 and 19 when compared with other women [9, 10].

In sub-Saharan Africa (SSA), adolescent ASRH has continued to take a back seat. Early marriage, teenage pregnancy, sexually transmitted infections (STIs), and high levels of unmet need for contraception are all common in many countries in the SSA region [11]. In Nigeria, teenage and unplanned pregnancy remains a major challenge, especially among adolescents. Hence, about 1.25 million commit induced abortion every year by unskilled providers and without recourse to post-abortion care, leading to complications [12]. Also, the percentage of adolescents who have begun childbearing increases from around 2% at age 15 to 37% at 19% [13]. Unmarried pregnant adolescents, particularly, face stigma and rejection, and some of them drop out of school [14, 15].

Evidence from some developing nations, including Nigeria, show that actions to make health services adolescent-friendly have led to increased uptake and participation of adolescents [16]. Hence, many countries have undertaken initiatives to make health services user-friendly and more appealing to adolescents, although, many of them are small in scale and highly limited in scope [17]. Nigeria has introduced youth-friendly services (YFS) in health institutions across the federation so as to improve access to ASRH [1]. The provision of adolescent-friendly health services plays a crucial role in the health and wellbeing of adolescents [18]. Such health services should be accessible, acceptable, equitable, appropriate and effective [19]. Thus, the World Health Organization (WHO) developed a guidebook for developing national quality standards for adolescent-friendly health services [20].

In many parts of southeast Nigeria, such as Ebonyi state, multiple stakeholders from government and non-government agencies have rallied to address the high teenage pregnancy, maternal mortality rates and other SRH challenges among adolescents in the State [21–23]. However, it remains unclear who is doing what and to what extent. This understanding is crucial, given that stakeholders play diverse roles in relation to any policy or program. Some may promote and support the policy or program, while others may try to hinder or frustrate the achievement of policy goals [24]. Besides, some may directly or indirectly influence policy design or program implementation.

A clearer picture of the activities of the various stakeholders involved in adolescent SRH services can be achieved through stakeholder mapping and analysis. This is necessary as most studies on adolescent health services in the State have focused mainly on the demand side of ASRH services [23, 25]. The stakeholder mapping and analysis approach will help identify and prioritize key ASRH stakeholders [26].

This paper contributes to information on the positions, roles and involvement of public institutions and NGOs, as well as on the degree of their influence, support or otherwise in adolescent SRH policy-making and program implementation. This will inform mechanisms for stakeholder engagement, advocacy, and resource allocation to priority areas for greater impact.

## Methods

### Study area

The study was carried out in Ebonyi state, one of the five states in the southeast geo-political zone of Nigeria. It is located on latitude: 6° 15′ 18“ N, longitude: 8° 05’ 55” E, and shares a border with Benue State to the north, Enugu State to the west, Imo and Abia States to the south and Cross River State to the east [27]. The State has thirteen local government areas (LGAs), six LGAs were purposively selected to represent the three senatorial zones and geographical locations (urban and rural) of the State, for the study. The selected LGAs were i) Afikpo South, ii) Abakaliki, iii) Ezza South, iv) Ikwo, v) Izzi, and vi) Ohaozara local government. A community was purposively selected from each local government area. The communities that the State government had underlined for strengthening adolescent SRH interventions were prioritized.

Ebonyi state has an estimated population of about 6 million inhabitants, and more than 40% of the State’s population is below 15 years of age [28]. About 9.6% of adolescent girls aged 15 to 19 have begun childbearing, and the maternal mortality ratio in this age group was reported as 30.5% in 2018 [21–23].

### Study design and study population

This qualitative research used an exploratory approach to collect detailed information on the involvement and support systems of adolescent sexual and reproductive health. The stakeholder mapping and analysis framework was used to identify the roles and involvements of stakeholders [29].

Information was collected through key informant interviews (KIIs) and focus group discussions (FGDs). A total of eighty-one (81) KIIs, comprising 25 state-level informants and 56 local government and community-level informants were conducted. Participants for the KII were policymakers, program managers and health service providers at the State and local government levels.

The state-level key informants were drawn from the State Ministry of Health (SMoH), State Ministry of Women Affairs and Social Development (SMoWASD), State Ministry of Youth and Sports (SMoYS), State House of Assembly (SHoA), State Ministry of Education (SMoE), State Universal Basic Education Board (SUBEB), State Primary Health Care Development Agency (SPHCDA), and Non-Governmental organization (NGOs).

The local government and community-level key informants included officers in charge (OICs) of PHCs, LGA adolescent health focal persons, patent medicine vendors, traditional rulers, religious leaders, town union chairman/president, ward development chairman, parents of adolescents aged 13-18 years, principals of public secondary schools and LGA administrative secretary.

A total of six (6) FGDs was, also, conducted. Each FGD comprised 8 to 11 participants, giving a total of 59 participants. The participants for the FGDs included community leaders/village heads, school authorities and parents of adolescents aged 13 to 18 years.

### Sampling technique

Random and purposive sampling techniques were used in the study. Six local government areas (LGAs), two from each senatorial zones, were selected from the thirteen local government areas in the State to ensure representation of geographical (urban and rural) and geopolitical locations. A community was selected from each of the local government areas, based on the high rates of teenage pregnancy and abortion, as recommended by the key stakeholders in the State Ministry of Health.

The key informants were purposively selected based on their participation in adolescent SRH, and to represent a wide range of values, cultures and religions. A starting list of public institutions and stakeholders involved in adolescent health was generated, and the list was expanded through referrals.

The participants for the FGDs were also purposively selected based on their leadership positions in the communities.

### Data collection

The data were collected using two interview guides that were developed by a team of qualitative researchers. The interview guides were pre-tested in a contiguous state. Before data collection, all participants were informed of the study’s objectives, and written consent was obtained.

The data were collected by trained qualitative researchers with skills and experience in conducting qualitative interviews. The FGDs were facilitated by a moderator, a note taker and a local guide/translator. All the interviews were audio recorded with the permission of the key informants and participants. The interviews and discussions were held in venues that were convenient for participants. KIIs were held in English language, while FGDs were held in the local language.

### Data analysis

The audio files were translated to English where necessary and transcribed verbatim. All transcripts were anonymously coded and stored in a laptop that is password protected. We adopted the stakeholder mapping analysis approach from Murray-Webster & Simon (2006) in which stakeholders are first described in terms of power, attitude and interest (stakeholder potentials). This is then followed by a mapping of their positionality of support or non-support [29].

**Murray-**Webster & Simon stakeholder mapping/analysis Framework (using stakeholder potentials and positions which are described below) [6, 29]:

#### Stakeholder potentials

According to the framework, stakeholder potentials are categorized based on three dimensions, namely

1. **Power**. This involves the perception of stakeholders about their influencing power on adolescent SRH program implementation in the State. This study categorized their influencing power as either ‘strong’ or ‘weak’.
2. **Attitude**. This involves stakeholders’ perception of how they support adolescent SRH program implementation in the State. This study categorized their support as either ‘supportive’ for positive attitude or ‘non supportive’ for negative attitudes.
3. **Interest**. This is the perception of stakeholders to engage in the adolescent SRH program implementation. Interest was categorized as either ‘active’ for those who have strong interest, and are willing to engage in the adolescent SRH program or ‘passive’ for those unwilling or reluctant to engage in the adolescent SRH program.

#### Stakeholder positions

This is a mapping of the stakeholders’ positions in providing adolescent sexual and reproductive health information and services in the State. It involves an analysis of the potentials (power, attitude, and interest) of each stakeholder (decision-makers, providers, and influencers) relating to their functions and roles in adolescent SRH policymaking and programming. The degree of interaction of the three dimensions of stakeholder potentials determines their positionality (support or non-support) and the specific category within the positions.

For the supportive stakeholders, the categories include [6, 29]:

- **Savior.** This is addressed to those with strong power, supportive attitude and active interest in adolescent SRH program. The parties will do whatever is necessary to maintain their position and try to attend to adolescent SRH needs.
- **Friend.** Refers to those with weak power, supportive attitude and active interest in the adolescent SRH program. They should be positioned as advisers.
- **Sleeping giant.** This refers to those with supportive attitude, strong power and passive interest in adolescent SRH program. In order to be awakened, this category of people should be included in the process.
- **Acquaintance**. This refers to those with supportive attitude, weak power and passive interest in adolescent SRH program. This category of stakeholders should be kept in touch, communicated and provided with continuous information.

For non-supportive stakeholders, the categories include, [6, 29]:

- **Saboteurs** are those stakeholders with non-supportive attitude, strong power and active interest.
- **Trip wire** refers to those who have non-supportive attitude, weak power and passive interest in adolescent SRH programs. They need to be understood, so that they can be carefully approached.
- **Irritants** are those with non-supportive attitude, weak power and active interest in adolescent SRH programs.
- **Time**-**bomb** refers to stakeholders with non-supportive attitude, strong power and passive interest in the adolescent SRH program.

## Results

### KII and FGD participants’ profile

For the KIIs, a total of 81 participants (25 at the state level and 56 at the LGA and community levels) that were interviewed. The participants were 42 males and 39 females; 53 were from urban and 28 were from rural areas. They included 31 policymakers or program managers, 19 health service providers, 17 community leaders, 6 religious leaders and 8 parents of adolescents.

For the focus group discussion (FGDs), a total of 59 participants were involved in six FGDs consisting of eight to eleven participants per session. Three FGDs each were done in the urban and rural communities. Most of the participants were farmers (41), while others were civil /public servants (5), retirees (4), artisans (3), traders (3), teachers and a priest.

### State-level policy/decision makers’ mapping based on their perceptions of their influence, attitude and interest in ASRH

As illustrated in Table 1, the identified state-level policy/decision makers consist of nine public/government institutions and five non-governmental organizations (NGOs) categorized as one institution. The potentials and positions of state-level decision makers are presented subsequently according to three main functions, namely: policy advocacy and development, program implementation and service delivery, and, lastly, technical support and capacity building.

**Table 1:** Mapping of State-level public/government institutions and NGOs

Public institutions /NGOs		Policy advocacy and policy development	Program implementation and service delivery	Technical support and capacity building
State primary healthcare development agency (SPHCDA)	Power	–	+	+
	Attitude	–	+	+
	Interest	- TRIP WIRE	+ SAVIOR	+ SAVIOR
SMOH (Public Health; Reproductive health unit; Gender; School health; Family planning; Social mobilization)	Power	+	+	+
	Attitude	+	+	+
	Interest	+ SAVIOR	+ SAVIOR	+ SAVIOR
DPRS (Dept. of Planning, Research and Statistics)	Power	+	–	–
	Attitude	+	–	–
	Interest	- SLEEPING GIANT	- TRIP WIRE	- TRIP WIRE
SMOE (Education Service dept.; School Service unit)	Power	+	+	–
	Attitude	–	+	+
	Interest	- TIME BOMB	+ SAVIOR	+ FRIEND
ESUBEB (Ebonyi State Universal Basic Education Board)	Power	–	+	–
	Attitude	–	+	–
	Interest	- TRIP WIRE	+ SAVIOR	- TRIP WIRE
Ministry of Women Affairs and Social Development (Gender unit; Social welfare unit)	Power	–	+	–
	Attitude	+	+	+
	Interest	- ACQUAINTANCE	+ SAVIOR	+ FRIEND
Ebonyi State Ministry of Youths and Sports	Power	–	–	–
	Attitude	–	+	+
	Interest	- TRIP WIRE	+ FRIEND	+ FRIEND
Ebonyi State House of Assembly (State Legislature)	Power	+	–	–
	Attitude	–	–	–
	Interest	+ SABOTEUR	- TRIP WIRE	- TRIP WIRE
Institute of Child Health, FETHA	Power	–	+	–
	Attitude	–	–	–
	Interest	- TRIP WIRE	+ SABOTEUR	+ IRRITANT
**NGOs** (AMURT; JHPEIGO; Safe Motherhood Ladies; Health Policy Plus; BAN)	Power	–	+	+
	Attitude	+	+	+
	Interest	+ FRIEND	+ SAVIOR	+ SAVIOR

*Power:* (+) Strong power/influence, (−) Weak power/influence; *Attitude* (+) Supportive attitude, (−) Not supportive attitude; Involvement/interest: (+) actively involve (has strong interest), (−) passively involve (has no interest)

### Policy advocacy and development

The results from the mapping of state-level public institutions and NGOs revealed that some of the public institutions had a strong support for adolescent sexual and reproductive health policies and programs, while some others showed only a passive support for adolescent SRH matters. Only the State Ministry of Health was categorized to be ‘savior’ because the identified departments (public health department, reproductive health unit, gender unit, school health unit, family planning unit and social mobilization unit) had strong power/influence, supportive attitudes and were actively involved in adolescent SRH policy advocacy and development.

*“Policies guide the activities of people practicing in the State. We follow the national guidelines and modified as necessary. We develop some policy documents; there is one we are just working on, it is not yet finalized.”* (SPM02-male _Public health department SMOH).

*“We are involved in developing annual work plan targeting adolescents and this is what guides us for the year. We use the 2006 School Health Policy.”* (SPM08-Fmale _School health unit SMOH).

*“*[I] *was involved in the roll-out of minimum care package for adolescent SRH as highlighted in the Adolescent SRH policy* [of] *2007 … All [ …*] *desk officers from the whole 36 states including FCT participated...”* (SPM05-Female _reproductive health unit SMOH).

The department of planning, research and Statistics was categorized as ‘sleeping giant’ as they had strong power/influence, a low interest, but a positive attitude to adolescent SRH policy advocacy and development.

*“...Strategic plans of all the activities in the ministry are embedded in our own strategic plan. So, adolescents’ [health] is well captured there.”* (SPM03-Male_budget and planning).

Amongst the nine public institutions, four institutions which includes, Institute of child health FETHA, State Ministry of youths and sports, the State Universal Basic Education Board (ESUBEB) and the State Primary Health Care Development Agency (SPHCDA) were categorized as ‘trip wire’ due to their less power/influence, a non-supportive and passive attitude on adolescent SRH policy advocacy and development. However, the NGOs were categorized as ‘friend’ because they had a supportive attitude, strong interest but with a less power/influence in developing adolescent SRH policies and advocacy. State ministry of education was categorized as ‘time bomb’ while ministry of women affairs and social development was categorized as ‘acquaintance’.

*“Our organization does not formulate policies, though the organization was part of the development at the beginning stages; and we also align with the national policy on adolescent SRH.” (*SPM23-Female _NGO).

*“*[My] *organization is not really or directly involved in policy formulation. Indirectly, we have been invited to planning meetings at the State Ministry of Health and by partners. I will say a little because we do not have the power to formulate policies except, we are called to be part of it. But, we also look out for times when we will be invited to fully participate in developing policies on adolescent SRH”* (SPM20-Female _NGO).

In both implementation of adolescent SRH program and engagement with local authorities on adolescent SRH, three institutions were categorized as ‘savior’ while another three institutions were categorized as ‘savior’ in implementation of adolescent SRH program only. The state ministry of health, SPHCDA and NGOs were categorized as ‘savior’ due to their strong power/influence, a supportive attitude and an active interest/involvement in the implementation of adolescent SRH program and engagement with local authorities on adolescent SRH. The ministry of education, ESUBEB, and ministry of women affairs and social development were categorized as ‘savior’ due to their active involvements/interests, supportive attitudes and strong power in implementation of adolescent SRH program. The ministry of youths and sports was categorized as ‘friend’ because of the institution’s active interest/involvement and supportive attitude in both implementation of adolescent SRH programs and engagement with local authorities.

The State-level public institutions described the implementation of adolescent SRH program and involvement with local authorities on adolescent SRH in various ways. They revealed that they implement one or two adolescent SRH programs which includes; adolescent SRH information and services, capacity building, and technical support to other institutions for adolescent SRH.

### Program implementation and service delivery

While some provided both information and services related to adolescent SRH, others participated at the level of only service provision or information dissemination. Most of the public institutions took part in sensitization, and awareness creation on the need for SRH services and sexuality education through the communities, schools or mass media. The content of such sensitization is designed to reflect the focus and the area of interest of the implementing institution. Example is the promotion of information against gender-based violence (GBV) through media campaigns by the Ministry of women affairs. Another role played by some institutions is guidance and counseling services either in school or at the health centers. Few institutions, including the SMOH, SPHCDA and NGOs are involved in the actual provision of contraceptive services to the adolescent. Here are quotes to support our claim;

*“Every year we incorporate adolescent SRH into our programs, and create strategic plan. Our involvement is mostly in sensitization. We are social welfare people, we render social services, counseling, psychosocial duties and referrals. For instance, if a girl becomes pregnant, it is a problem. We emphasize the need for the girl child to have information on how to prevent rape and STI. We go to schools and sensitize them on female genital mutilation (a health factor) which is rampant in Ebonyi State.”* (SPM17-Female_Ministry of women affairs).

*“The reproductive health unit of the Ministry of Health is doing well as it concerns our mothers, children and even adolescents because in that unit, we have family planning as one of the components and adolescents require family planning which is the contraception we are talking about. So, the Ministry of Health is assisting in that area to ensure that the adolescents receive this contraception.”* (SPM05-Female _reproductive health unit SMOH).

*“We go on family planning sensitization. We also go on advocacy visits to the opinion leaders in the community because they are highly regarded in the society. We educate them on the implications of teenage pregnancy; that when a teenage girl has so many children, it poses a lot of challenges to the society”* (SPM09-Female_social mobilization unit SMOH).

*“We have service delivery partners who provide commodities. Our target is to change the social norms of child spacing, by engaging the traditional leaders, community leaders and the religious* [leaders] *to address barriers to contraception through communication tools that we developed. We identify upstanding members of the community and use them to reach out to the members of a community.”* (SPM 21- Female _NGO).

*“The Primary Health Care Development Agency is fully involved in adolescents’ reproductive health in the sense that we have created a budget line for procuring contraceptives and these contraceptives have been procured. They will be made available to the adolescents to prevent unwanted pregnancies and sexually transmitted diseases. In fact, to tell you how important it is, we have focal person that is responsible for reproductive issues, we call them sexual and reproductive health focal person. So, it is a very big priority to the agency.”* (SPM01-Male_SPHCDA).

Activities are made available in youth friendly environments to encourage adolescents have access to them and to feel free in disclosing sensitive information. Some of the institutions are involved in the establishment of youth friendly centers while others provide their services in already existing structures/facilities. Still others do not establish or use physical structures but provide platforms that encourage adolescents to interact with one another and adopt healthy sexual and reproductive health attitudes and behaviors. Examples of such platforms are peer clubs set up in schools which create an opportunity for activities, interaction and formation of healthy relationships among adolescents.

*“Safe motherhood* [initiative] *created a youth center where adolescents are invited sometimes with their parents for sensitization programs.”* (SPM23 female, Safe Motherhood Ladies Association).

*“*[We have] *a drop-in center for out-of-school adolescents to access information on SRH while acquiring vocational skills”* (SPM16_ Women Affairs and Social Development).

### Technical support and capacity building

Two departments in the SMOH, including the School Health and the Reproductive Health units demonstrated how they have been actively involved in training teachers, head teachers, and trainers. The capacity of selected teachers in secondary schools are built to enable them teach sex education appropriately. Also, principals, head teachers, education secretaries are trained on sex education so they can (step down) take it back to their locations. Furthermore, capacity building was noted to occur in form of empowering adolescents with relevant skills, and giving small business start-up loan.

*“The Ministry of Health also builds the capacity of these adolescents by organizing trainings for them to pass this information concerning them. All these issues we have enumerated, issues about STI, issues about contraception, issues about malaria and so many other programs. So, they organize trainings for them, both for their providers and for the adolescents themselves.”* (SPM05-Female _reproductive health unit SMOH).

*“The Ministry has trained over 80% of their health workers on adolescent health” (*SPM08- Female _school health unit SMOH).

Some of the institutions, such as SMOE and the Ministry of Women affairs, give support either directly to adolescents or do so in collaboration with some State public health institutions or development partners. For instance, in assisting and re-integrating victims of sexual violence and rape, and the economic empowerment of unwedded mothers, particularly, adolescents, some of these institutions carried out their activities in collaboration with the SMOH, Maternal and Child Survival Program (MCSP), UNICEF, or UNFPA as necessary. Also, it was indicated that a non-governmental organization, John Hopkins Program for International Education in Gynecology and Obstetrics (JHPIEGO) has supported the State public health institutions in ASRH programs. Below are some supporting quotes;

*“Well, we have supported the State to strengthen the adolescents’ sexual* [and] *reproductive health. We also work closely with the Ministry of Health, the adolescent focal person and desk officer. We work closely with her and her team in all these things I have said”* (SPM 24- Female _NGO).

*“[We] collaborated with MOH, UNICEF and UNFPA in training teachers and peer educators on how to provide SRH information to students”* (SPM 12- Female _SMOE).

*“In collaboration with the media house, they* [media] *send out jingles* [to] *sensitize parents, communities* [and] *adolescents, and* [to] *inform the public* [of] *where they can access adolescent counseling services”* (SPM 15-Female_Ministry of Women Affairs).

### Local government decision makers’ and community leaders’ mapping based on their perceptions of their influence, attitude and interest in ASRH

As shown in Table 2, the local government decision makers and community influencers consisted of six institutions/ offices which include; traditional rulers and village heads, community religious leaders, adolescent SRH focal persons, town union and ward development chairmen, community public schools, and local government administrative secretaries. The potentials and positions of the stakeholders are presented subsequently according to the three main functions: policy advocacy and development, service delivery and program implementation, and technical support and capacity building.

**Table 2:** Mapping of local government (LGA) level decision makers and community influencers

LGA/Community influencers and Service Providers		Policy advocacy and development	Program implementation	Engagement with local authorities and organizations
Traditional Rulers and Village Heads	Power	–	+	+
	Attitude	+	–	+
	Interest	+ FRIEND	+ SABOTEUR	+ SAVIOR
Religious organizations and leaders	Power	–	+	+
	Attitude	–	+	–
	Interest	- TRIP WIRE	- SLEEPING GIANT	+ SABOTEUR
Adolescent and reproductive health focal persons (LGA)	Power	–	+	–
	Attitude	–	+	+
	Interest	- TRIP WIRE	+ SAVIOR	+ FRIEND
Public Secondary Schools (Principals & Guardian counsellors)	Power	–	+	–
	Attitude	+	+	+
	Interest	- ACQUAINTANCE	+ SAVIOR	+ FRIEND
Town Union and Ward Development Chairmen	Power	–	–	+
	Attitude	–	+	+
	Interest	- TRIP WIRE	+ FRIEND	+ SAVIOR
LGA Administrative Secretaries	Power	–	+	–
	Attitude	+	+	+
	Interest	+ FRIEND	+ SAVIOR	+ FRIEND

*Power:* (+) Strong power/influence, (−) Weak power/influence; *Attitude* (+) Supportive attitude, (−) Not supportive attitude; Involvement/interest: (+) actively involve (has strong interest), (−) passively involve (has no interest)

### Policy advocacy and development

In policy advocacy and development, most institutions/offices at the local government level were categorized as ‘trip wire’. The office of traditional rulers/village heads and local government administrative secretaries were categorized as ‘friend because of their interests and supportive attitudes in policy development and advocacy but, weak powers towards the strategy. The office of traditional rulers/village heads and local government administrative secretaries opined that they either develop community by-laws or adapt federal and State level policy to forms the local government by-laws which govern their activities on adolescent SRH.

*“I would have liked it if I am called to be part of the policy* [development for adolescent SRH] *... We used to have a program where we go to develop the policy that will be guiding reproductive health* [programming] *in the State... From the national policy, we develop the state policy.* [And] *from the state policy we develop the local government policy. It is the backbone from where we step down to the communities …* “(IIK01- Male_ administrative secretary).

*“*[I am] *not part of it* [policy development for adolescent SRH]. *But, in our community, we made a law that if you impregnate a girl, you will marry her. But if you say you will not marry then you will have to stay with the person until she delivers the baby and the baby starts walking then you can let her go. You will take care of the girl* [adolescent] *and the baby. And you, the male, after going through the suffering it will be better for you to marry her. That is the law of the community and in having this type of policy the Igwes* [traditional rulers] *should be called to say their mind.”* (VHEZ- Male_ village head).

The public schools were found to be ‘acquaintance’ as they had supportive attitudes with weak powers and passive interests in policy advocacy and development. A principal categorically opined that their role in policy development is to generate evidence for policy makers:

*“We generate the information and give to them for developing* [policy]; *though information goes two ways – it is either from the top to down or down to top. Initially, there is nothing like sex education in secondary schools. We then discovered with AIDS and all what not, that it is necessary to* inculcate [sex education] *in the scheme of work. We now prepared the scheme of work and sent to government; government approved it and returned to us to implement”* (IIZ01- Male_ school principal).

### Program implementation and service delivery

Concerning implementation of adolescent SRH programs, public secondary schools, LGA administrative secretaries and LGA focal persons on adolescent SRH were categorized as ‘savior”, while town union/ward development chairmen were found to be ‘friend’.

*“...We have the office of the guidance counselor and students go and they talk privately. We also do sex education through the guidance counselor or health personnel that visit the school. We provide information on reproductive health system, secondary sexual characteristics of boys and girls, puberty and others”* (IEZ01-Male_School principal).

*“...Yes, as the health department in* [the] *LGA, we embark on sensitization program for adolescents in schools, and in health facilities. On our outreach days, we call on teenagers* [and] *their parents and educate them on what they should be doing. We do this every Tuesday; and also during immunization, we give health talks on adolescent sexual and reproductive health for teenage mothers to benefit. Also, we do carry out a special information campaign on adolescent SRH with people within the age of 15 to 49 for healthy living”* (IOH04-Female_LGA focal person on adolescent SRH).

The religious leaders had strong power/influence and supportive attitude towards implementation of adolescent SRH programs. However, they were found to have passive interest/involvement in the programs. The office of traditional rulers/ village heads and town union/ ward development chairmen were categorized as ‘savior’ in engagement with local authorities on adolescent SRH.

*“We have the youth organ in the church; and as a teacher, once in a while we make arrangements for them to have some kind of seminar where we teach them about being young people and growing into adulthood. And in such seminars and retreats, they are being exposed to things they need to know about their sexuality*.*”* (IIZ02-Male catholic religious leader).

*“Here also, in the school, all of them are adolescents. So, we find time and teach them out of class. As I told you, the* [guidance] *and counseling (G&C) teacher is transferred almost every section, and a new person is posted to the school. The G&C will take care of the student by guiding and counseling them on adolescent SRH. It is through this* [guidance] *and counseling that you will know the problem of individual students. Occasionally, we give them talks on reproductive health. Like every Friday, we have social gathering and we channel it* [the talk] *on reproductive health so that they* [students] *will know the dos and don’ts”* (IIZ01- Male_ School principal).

Among those who were categorized to be ‘savior’ programs on adolescent SRH were implemented in various ways, including, adolescent SRH information and services, capacity building and collaboration with other institutions to achieve their objectives.

*“We employ some experts, recruit and train some people and post them to different places where we provide those (SRH) services”* (IIK01- Male_ administrative secretary).

*“We have been receiving some NGOs. Some health worker have been coming to educate students mostly on Thursdays when we usually have our moral instructions. We do welcome some of them when they come to educate the students. We also appreciate their efforts because they are helping us to do our work”* (IAB02- Female_ School principal).

### Engagement with local authorities and organizations

In the area of engagement with local authorities, traditional rulers/village heads and town union/ward development chairmen were classified as “savior”, while, public secondary schools and admin secretaries of local government areas were viewed as “friends”. Religious organizations and leaders were regarded as “saboteur”, based on their non-supportive attitude, strong influence and active interest.

*“At the beginning of a new session, we organize an event called, “come and hear“. It is a time the villagers come to hear what the school intends to do about the SRH issues, such as teenage pregnancy, that affect the girl child. We carry everybody along. So, before the event, we meet with relevant authorities in the village and engage with them.”* (IIZ01-Male_principal).

*“...we engage community mobilization officers, and the traditional institutions in order to mobilize people and educate them.”* (IIK01-Male_Admin sec., LG Health Department).

“What has been helpful to me is the experience I gathered over the years as a priest, and the support from the traditional authorities, such as traditional rulers, village heads, parents and other village members because of my active engagement with these groups.” (IIZ02- Male catholic religious leader).

### Discussion

This study explored the positions, roles and involvements of public institutions and NGOs in ASRH policy making processes and program implementations in Ebonyi State, southeast, Nigeria. The results of this study shsow that the majority of identified government and non-government stakeholders participate actively in ASRH, albeit to differing degrees and dimensions. However, some important stakeholders only take a peripheral interest in ASRH policies and activities. As regards the involvement of public institutions in policy advocacy and policy development, findings show that the State Ministry of Health was the only institution categorized as ‘savior’ at the state level, because the identified departments (public health department, reproductive health unit, gender unit, school health unit, family planning unit and social mobilization unit) had strong power/influence, supportive attitude and are actively involved in ASRH policy advocacy and development. This is consistent with findings from studies carried out in other countries, such as Bangladesh [30] and Indonesia [6]. It is encouraging that the State Ministry of Health is committed and actively involved in increasing adolescents’ access to SRH information and services through adolescent SRH program advocacy and development. As part of that commitment, the ministry assumes a leading role in ASRH program advocacy, as well as adapt national policies relating to adolescent SRH to the state context as observed in this study. However, it is worrisome that, at that level, only the ministry of health was identified as ‘savior’, while the majority of the public institutions were in the position of ‘trip-wire’. This is a shortfall in the development of ASRH policies in the State because the development process for an effective ASRH framework (policy) should be participatory with inputs from a wide range of stakeholders. This is of critical importance as it helps to recognize the interests and local values of the various stakeholders.

In the same vein, it is unfortunate that the legislature (the State House of Assembly), which should be in the forefront of making appropriate legislations in support of ASRH was identified, in our study, as ‘saboteur’ in relation to their involvement in ASRH policy advocacy and development. The legislature was indicated as powerful, with high interest, but negative attitude towards ASRH policy advocacy and development. Given their status as one of the major arms of the State government, more intensive advocacy to them should be a priority [29]. It is important that they are engaged and convinced about the part that they can play in this crucial endeavor. In addition, the department of Planning, Research and Statistics (DPRS) was identified as ‘sleeping giant’. The department is concerned with routine collection, analysis and interpretation of health data for decision making; setting goals, prioritization and formulation of health policies for the State health sector [31]. This empowers them to influence resource mobilization and allocation for activities in the health sector. Thus, there is, also, the need for strong advocacy to the department to ensure the integration of ASRH issues into the development planning and provision of adequate budgetary allocations for ASRH activities. However, in contrast to this finding, a study in Indonesia showed that a similar department (the Regional Planning and Development Board) had a strong influence, supportive attitude and positive interest in ASRH policies and programs, and was, therefore, classified as a ‘savior’ [29]. This difference in findings might be a result of different levels of policy-maker support for ASRH policies in the respective countries.

Also, bothersome is the finding that the state universal basic education board, was categorized as a ‘trip-wire’, which means that they had low power, low interest, and negative attitude. Considering the role of school in the promotion and protection of sexual and reproductive health of adolescents, it is important that they are understood and actively engaged with. Whereas their role in policy development can be limited, their interest in advocacy should be better than was observed in this study; they should be involved in ASRH policy advocacy and formulations. Additionally, there is need for synergies and partnership between health and education sectors in the areas of ASH policy-making and program implementation in view of the widespread nature of schools. Such collaborative partnerships are essential for improved health and education outcomes [32]. Moreover, successful implementations of adolescent SRH programs will depend on close collaboration and partnership among various sectors, with the health sector leading and coordinating the processes [1].

On another note, it is heart-warming that more than half of the state-level public institutions were identified as ‘savior’ with regards to the implementation of adolescent SRH programs in the State. It is also worthy of note that NGOs were identified as ‘savior’ in this regard. This corroborates the evidence that NGOs, with support from donor agencies, are active in adolescent SRH, and have been implementing programs and interventions that address ASRH issues within and outside Nigeria [33–35]. In the study area, various NGOs with different priorities have responded to the SRH needs of adolescents. The result is, also, in keeping with findings from previous studies in other countries, such as Tanzania and Malaysia which documented the remarkable contributions by NGOs to ASRH program implementations and sustainability [36, 37]. Hence, it is not surprising that NGOs, along with the State Primary health Care Development Agencies (SPHCDA) and the State MOH were identified as ‘savior’, in this study, in the area of technical support and capacity building.

At the local government level, most institutions and offices were categorized as ‘trip wire’ in policy advocacy and development due to their weak power/influence, unsupportive attitude and passive interest or involvement in advocacy and development of policy. The office of traditional rulers/village heads was, however, categorized as ‘friend’ because of their positive interest, supportive attitude, but weak power in relation to policy development and advocacy. This result is consistent with findings from previous studies in other parts of the SSA, where traditional institutions employed by-laws, fines and other forms of penalties, as also observed in this study, to influence health behaviors in the communities [38, 39]. For this reason, traditional leaders have been described as key stakeholders in the fight against HIV and promotion of good health in some communities [38]. Thus, to gain the support of a community, there is need for advocacy and awareness creation among stakeholders at the local level, who are the gatekeepers. Engaging these significant others, right from program design, has been shown to promote trust and respect towards such programs [40]. It facilitates the implementation of strategies for delivering SRH services and information to adolescents, through their institutionalization and adoption in communities [5].

Engagement with local authorities follow a defined process to identify and respond to perceived local drivers of and barriers to sexual and reproductive health [41]. These are ‘high-impact practices’ which help to understand SRH issues from the perspective of the community, and should be promoted widely [41]. In this regard, the offices of traditional rulers, town Union and Ward Development Chairmen (WDC) were given the position of ‘savior’ in our study, while, religious institutions/leaders were considered ‘saboteur’. This is because of their strong power, and active interest in ASRH matters, but non-supportive attitude to engaging local authorities on such matters. Religious leaders were, also, categorized as ‘sleeping giants’ and ‘irritants’ regarding their positions in ASRH policy-advocacy and program implementation, and this calls for concern from policy-makers and public health actors. This is in view of the popularity of religion among most African communities, and the influence religious leaders have over their adherents on such issues. Thus, it is important that they are actively engaged to harness the enormous power at their disposal in favor of adolescent SRH and wellbeing.

In implementation of ASRH programs, public secondary schools and local government stakeholders were categorized as ‘savior’, while town union and ward development chairmen were found to be ‘friend’. The role of public schools in supporting the implementation of ASRH programs is well-documented in earlier studies in both developed and developing countries [42–44]. School-based SRH education is one of the most important and common ways to help improve the reproductive health outcomes of adolescents [45]. For instance, comprehensive sexuality education (CSE) is integral to ensuring that adolescents in schools get the information they need to achieve healthy sexual and reproductive lives and avoid negative health outcomes. To this end, some government authorities have demonstrated commitments to CSE by implementing programs, which reflect the needs of adolescents in schools [44].

This study is not without some limitations. First, the non-random selection of participants may have led to selection bias, and so, the study findings may not be generalized to the entire stakeholders in Ebonyi state. Also, the sensitive nature of the topic could have led to information bias as our people often find it uncomfortable discussing sex-related matters. In addition, the involvement of local guides in the data collection process may, also, have limited in-depth exploration of information among participants in the communities. However, in spite of these limitations, the use of KII and FGD enabled an in-depth exploration of powers, interests and involvements of the various ASRH stakeholders in the State. In addition, the study used an analytical framework (Murray-Webster & Simon stakeholder mapping/analysis Framework) to classify the stakeholders according to their identified powers, attitudes and interests.

Future research should explore why certain stakeholders such as the State Ministry of Health showed stronger support to ASRH policies and programs, compared to stakeholders, such as the State legislature, Ministries of youth and sports, and education. Such studies should, also aim to unravel the drivers of these diverse interests, positions, and actions, as well as investigate the extent and effectiveness of collaborations between these stakeholders. This will provide more information for designing effective interventions in order to address the identified gaps.

## Conclusion

This stakeholder mapping has demonstrated that key stakeholders appear to play supportive roles in the implementation of ASRH programs in Ebonyi State, but many of the relevant government and non-government institutions are not involved in the policy-making process. There is a need for more intentional and active involvement of relevant stakeholders in policy-making, as this would contribute to better ownership and sustainability of ASRH interventions. It is, also, important that stakeholders that show only passive interests to ASRH policies and programs are engaged, in order, to improve ASRH programming in the State. The findings from this analysis would inform strategies for future stakeholder engagement. Thus, these findings can be used to prioritize engagement activities among the identified stakeholders and as well, plan for efficient allocation of limited resources.

Additionally, the study contributes to health policy research as it offers results that could inform future policy direction and engagement in the field of ASRH. Some powerful institutions, such as the legislature, and the DPRS that were identified as ‘saboteur’ and ‘sleeping giant’ respectively, need to be engaged and convinced about the roles that they can play in this important endeavor. Other stakeholders that should be prioritized for active engagement are the Ministry of Education and institute of child Health.

Continuous advocacy to state level policymakers is needed to secure and ensure political commitment, and mobilization of resources for adolescent health programming. Furthermore, there is need for advocacy and awareness creation among community gatekeepers such as parents, traditional rulers and religious leaders to increase their involvement in ASRH policy advocacy and program implementation.

## Data Availability

The dataset used for this study is available and can be obtained from the corresponding author upon request.

## Abbreviations

ASRH: Adolescent Sexual and Reproductive Health
ESUBEB: Ebonyi State Universal Basic Education Board
FGD: Focus group discussions
GBV: Gender-based violence
IDI: In-depth Interview
IDRC: International Development Research Centre
ICPD: International Conference on Population and Development
MOH: Ministry of Health
SRH: Sexual and Reproductive Health
SRHR: Sexual and Reproductive Health Rights
STIs: Sexually transmitted infections
SPHCDA: State Primary Health Care Development Agency
SMoWASD: State Ministry of Women Affairs and Social Development
SMOYS: State Ministry of Youth and Sports
SOME: State Ministry of Education
YFS: Youth-friendly services
